# Polatuzumab vedotin, rituximab, cyclophosphamide, doxorubicin, and prednisone (Pola-R-CHP) therapy in diffuse large B-cell lymphoma in patients aged 80 years or older: a real-world study

**DOI:** 10.1007/s00277-025-06619-0

**Published:** 2025-10-10

**Authors:** Toshiki Yamada, Nobuhiko Nakamura, Hideko Goto, Takeshi Hara, Senji Kasahara, Kenji Fukuno, Michio Sawada, Hiroshi Nakamura, Naoki Hayase, Takayuki Goto, Tomomi Suzaki, Kei Fujita, Shin Lee, Nobuhiro Kanemura, Masahito Shimizu, Hisashi Tsurumi

**Affiliations:** 1 Department of Hematology, Gifu Prefectural General Medical Center, Gifu, Japan; 2 Department of Hematology and Infectious Disease, Gifu University Hospital, Gifu, Japan; 3 Department of Hematology, Chuno Kosei Hospital, Gifu, Japan; 4 Department of Hematology and Oncology, Matsunami General Hospital, Gifu, Japan; 5 Department of Hematology, Gifu Municipal Hospital, Gifu, Japan; 6 Department of Hematology, Japanese Red Cross Takayama Hospital, Gifu, Japan; 7 Department of Hematology, Japanese Red Cross Gifu Hospital, Gifu, Japan

**Keywords:** Diffuse large B-Cell lymphoma, Polatuzumab vedotin, Pola-R-CHP, Geriatric oncology, Chemotherapy dose intensity

## Abstract

Diffuse large B-cell lymphoma (DLBCL) predominantly affects older adults, with an increasing proportion of patients aged ≥ 80 years. Although the POLARIX trial demonstrated improved progression-free survival with polatuzumab vedotin (Pola) combined with R-CHP (Pola-R-CHP), its applicability to very elderly patients remains unclear. A multicenter, retrospective, cohort study was conducted at seven hospitals in Japan, enrolling 172 previously untreated DLBCL patients who initiated Pola-R-CHP between August 1, 2022 and May 1, 2024. Patients were stratified by age (< 80 vs. ≥80 years). Initial dose intensity (IDI), average relative dose intensity (ARDI), treatment efficacy, and safety outcomes were evaluated. In patients aged ≥ 80 years (*n* = 44), the overall response rate (ORR) was 89.5%, comparable to the 97.3% in patients aged < 80 years. However, treatment-related mortality (TRM) was higher in the older group (11.4% vs. 2.3%). In this cohort, median IDI and ARDI for doxorubicin and cyclophosphamide were markedly lower, whereas Pola dosing was relatively preserved. Higher Pola IDI was associated with an increased incidence of severe adverse events (sAEs), and a lower Geriatric Nutritional Risk Index (GNRI) was independently correlated with both reduced ORR and increased sAEs. Pola-R-CHP demonstrates promising efficacy in DLBCL patients aged ≥ 80 years. However, the increased TRM highlights the importance of cautious toxicity management. Individualized dose modifications, particularly for cytotoxic agents, and comprehensive nutritional assessments may improve treatment tolerability and outcomes in this vulnerable population.

**Supplementary Information:**

The online version contains supplementary material available at 10.1007/s00277-025-06619-0.

## Introduction

 Diffuse large B-cell lymphoma (DLBCL) is the most common subtype of non-Hodgkin lymphoma and predominantly affects older adults, with the median age at Diagnosis exceeding 65 years worldwide [1]. In Japan, the proportion of patients aged ≥ 80 years with newly diagnosed DLBCL has been increasing in parallel with rapid societal aging [2]. Treatment of elderly patients remains a clinical challenge, since comorbidities, frailty, and organ dysfunction frequently necessitate dose modifications [3]. In particular, in patients aged ≥ 80 years, the standard regimen of rituximab plus cyclophosphamide (CPA), doxorubicin (DXR), vincristine, and prednisone (R-CHOP) is often associated with excessive toxicity, including febrile neutropenia, treatment-related hospitalization, and mortality [4]. Therefore, individualized treatment strategies that consider both efficacy and tolerability are essential in this vulnerable population.

Recently, the randomized, phase III, POLARIX trial demonstrated that substituting vincristine with polatuzumab vedotin (Pola) in the R-CHOP regimen, resulting in Pola-R-CHP, led to superior progression-free survival (PFS) without compromising safety [5]. However, the trial enrolled a relatively fit population, with a median age of 65 years, and excluded patients with significant frailty or poor performance status. Thus, data on the use of Pola-R-CHP in very elderly persons, particularly those aged ≥ 80 years, are scarce. In real-world practice, physicians often modify chemotherapy dose intensity to reduce toxicity risk. Still, little is known about how such modifications affect treatment outcomes with Pola-R-CHP in older adults [6, 7].

This study aimed to evaluate the real-world efficacy and safety of Pola-R-CHP in previously untreated DLBCL patients aged ≥ 80 years. In addition to characterizing age-related differences in initial dose intensity (IDI) and average relative dose intensity (ARDI), their associations with treatment response and severe adverse events were examined. By focusing on this underrepresented but clinically important subgroup, the findings provide insights into optimizing chemotherapy delivery and risk stratification for very elderly patients in routine practice.

## Materials and methods

### Study design and population

This was a multicenter, retrospective, cohort study conducted across seven hospitals affiliated with the Gifu Hematology Study Group. All consecutive adult patients (aged ≥ 18 years) with histologically confirmed, previously untreated DLBCL who started first-line treatment with Pola-R-CHP between August 1, 2022 and May 1, 2024 were included. Patients were excluded if they had primary central nervous system lymphoma, transformed indolent lymphoma, or a history of prior systemic therapy for lymphoma. This study was approved by the Ethics Committee of Gifu University Graduate School of Medicine (approval number: 2022 − 221), and the requirement for informed consent was waived due to the retrospective nature of the study and use of anonymized data.

### Clinical data and outcomes

Clinical, demographic, and laboratory data were extracted from medical records. The collected variables included age, sex, Eastern Cooperative Oncology Group performance status (ECOG PS), lactate dehydrogenase (LDH), Ann Arbor stage, number of extranodal sites, presence of bulky disease, soluble interleukin-2 receptor (sIL-2R), serum albumin, and International Prognostic Index (IPI) score [8]. Patients were dichotomized into two age groups: <80 years and ≥ 80 years. The cutoff of 80 years was pre-specified based on clinical relevance, since patients aged ≥ 80 years are generally considered “very elderly” in lymphoma studies and clinical practice, and previous reports have demonstrated Distinct treatment tolerability and outcomes in this population. In addition, the pivotal POLARIX trial included patients up to 80 years of age, making this threshold clinically relevant for comparison. Nutritional status was assessed using the Geriatric Nutritional Risk Index (GNRI), which was calculated as (1.489 × serum albumin [g/L]) + (41.7 × present weight/ideal body weight) [9]. Cell-of-origin classification was determined according to the Hans algorithm and categorized as either germinal center B-cell (GCB) or non-GCB subtypes [10].

The primary endpoint was the overall response rate (ORR), defined as the proportion of patients achieving complete response (CR) or partial response (PR) per the Lugano 2014 criteria [11]. Secondary endpoints included the CR rate, incidence of adverse events (AEs) graded according to the Common Terminology Criteria for Adverse Events (CTCAE) version 5.0 [12], and treatment-related mortality (TRM). Treatment efficacy was assessed at the end of therapy using positron emission tomography-computed tomography, and safety evaluations focused on TRM. In addition, potential predictors of treatment response and severe AEs (non-hematological grade ≥ 3) were examined using multivariable logistic regression analysis.

### Treatment regimen and dose intensity assessment

All patients received the Pola-R-CHP regimen, consisting of intravenous rituximab at 375 mg/m^2^, cyclophosphamide (CPA) at 750 mg/m^2^, doxorubicin (DXR) at 50 mg/m^2^, and Pola at 1.8 mg/kg on day 1, combined with oral prednisone at 100 mg/day for 5 consecutive days. This regimen was administered every 21 days for up to six cycles, with additional cycles 7 and 8 of rituximab monotherapy. Dose modifications and delays were made at the discretion of the treating physician based on clinical judgment and patient tolerance.

Relative dose intensity (RDI) for each drug was calculated as the ratio of the delivered dose intensity (mg/m² per week) to the planned dose intensity, expressed as a percentage. The average RDI across the regimen was calculated for each patient. In addition, two parameters were assessed for DXR, CPA, and Pola. Initial dose intensity (IDI) was calculated from the RDI in the first cycle, which reflects physician decision-making at treatment initiation and is often influenced by frailty and perceived tolerance. Average relative dose intensity (ARDI) was calculated as the mean RDI across all cycles, which reflects the sustainability of dosing and cumulative adjustments due to toxicities or complications. Rituximab and prednisone were not included in IDI and ARDI calculations. Where indicated, the mean IDI and ARDI across DXR, CPA, and Pola were used to summarize overall chemotherapy intensity. Distribution patterns of IDI and ARDI were visualized using kernel density plots stratified by age group.

### Statistical Analysis

Descriptive statistics were used to summarize patient characteristics. Continuous variables are expressed as medians with ranges, and comparisons between groups were made using the Mann–Whitney U test. Categorical variables were analyzed using the chi-squared test or Fisher’s exact test, as appropriate. To identify factors independently associated with treatment response and severe AEs, multivariable logistic regression models were constructed. The variables included were based on clinical relevance and prior literature. Odds ratios (ORs) and 95% confidence intervals (CIs) were calculated, and p-values < 0.05 were considered significant. All statistical analyses were performed using R version 4.2.2 (R Foundation for Statistical Computing, Vienna, Austria). During the preparation of this manuscript, the authors used AI language models, including ChatGPT-4o (OpenAI) and Gemini 1.5 Pro (Google), to assist with manuscript formatting and editing for language and style in accordance with journal guidelines. The authors have reviewed and edited the content and take full responsibility for the final version of the manuscript.

## Results

### Patient Population and Baseline Characteristics

A total of 172 patients with previously untreated DLBCL were included in this analysis, of whom 128 (74.4%) were aged < 80 years, and 44 (25.6%) were aged ≥ 80 years (Table 1). The median age of the overall cohort was 73 (range, 31–93) years. There were no significant differences between the age groups in sex, poor performance status (ECOG PS ≥ 2), elevated LDH, advanced Ann Arbor stage, involvement of ≥ 2 extranodal sites, bulky disease, or levels of sIL-2R. Although not significant, patients aged ≥ 80 years tended to have lower serum albumin (35 vs. 37 g/L, *p* = 0.081) and GNRI (93.6 vs. 95.3, *p* = 0.068), suggesting a trend toward impaired nutritional status in the older group. The distribution of IPI risk categories and Hans algorithm-defined cell-of-origin subtypes was also similar between the groups (*p* = 0.125 and *p* = 0.493, respectively), with comparable proportions of GCB and non-GCB subtypes.

**Table 1:** Baseline characteristics of patients stratified by age group (<80 years vs ≥80 years)

	Age <80 y(n = 128)	Age ≥80 y(n = 44)	P-value
Median age, y (range)	70 (31-79)	83 (80-93)	<0.001
Male, n (%)	77 (60.2)	22 (50.0)	0.289
ECOG PS ≥2, n (%)	17 (13.3)	10 (22.7)	0.153
LDH >UNL, n (%)	84 (65.6)	34 (77.3)	0.188
Ann Arbor stage ≥3, n (%)	83 (64.8)	28 (63.6)	0.999
Extranodal sites ≥2, n (%)	52 (40.6)	15 (34.1)	0.695
Bulky mass, n (%)	31 (24.2)	10 (22.7)	0.999
sIL-2R, U/mL, median (range)	1583(223-60568)	1663(598-29936)	0.636
Albumin, g/L, median (range)	37 (20-47)	35 (20-45)	0.081
GNRI, median (range)	95.3(64.1-110.2)	93.6(66.1-108.7)	0.068
IPI category, n (%)			0.125
Low	27 (21.1)	3 (6.8)	
Low-intermediate	37 (28.9)	12 (27. 3)	
High-intermediate	25 (19.5)	12 (27.3)	
High	39 (30.5)	17 (38.6)	
Hans algorithm, n (%)			0.493
GCB	61 (47.7)	24 (54.6)	
Non-GCB	61 (47.7)	17 (38.6)	
Unknown	6 (4.6)	3 (6.8)	

Abbreviations: ECOG PS, Eastern Cooperative Oncology Group Performance Status; LDH, lactate dehydrogenase; UNL, upper normal limit; sIL-2R, soluble interleukin-2 receptor; GNRI, Geriatric Nutritional Risk Index; IPI, International Prognostic Index; GCB, germinal center B-cell; Non-GCB, non-germinal center B-cell

### Dose Intensity and Treatment Modifications

**Fig. 1:**
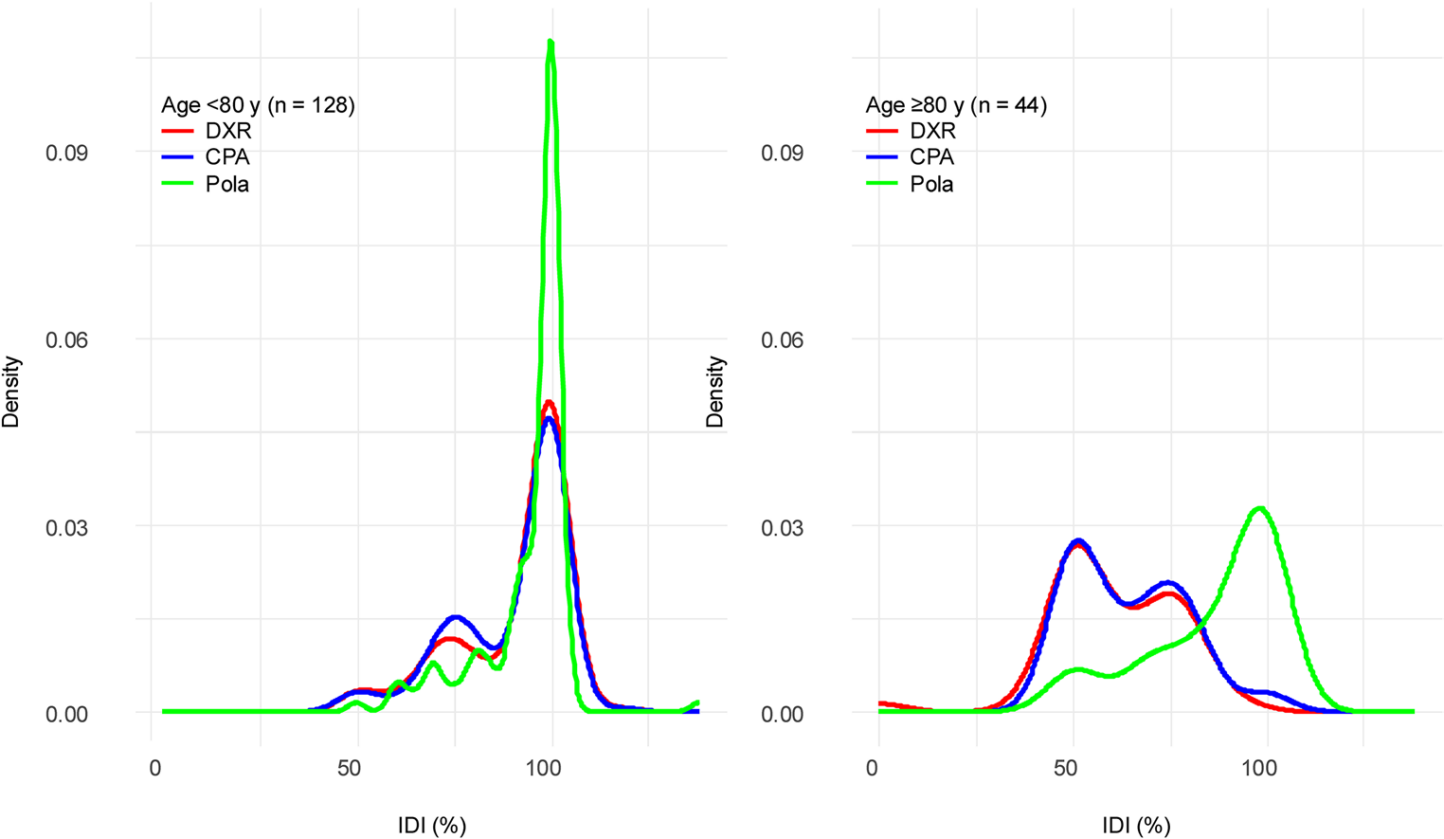
Density plots of initial dose intensity of DXR, CPA, and Pola by age group (< 80 years vs. ≥ 80 years) Kernel density plots showing the distribution of initial dose intensity (%) for doxorubicin (**DXR**), cyclophosphamide (**CPA**), and polatuzumab vedotin (**Pola**) in patients with diffuse large B-cell lymphoma. The left panel represents patients aged < 80 years (n = 128), and the right panel represents those aged ≥ 80 years (n = 44). Abbreviations: IDI, initial dose intensity; DXR, doxorubicin; CPA, cyclophosphamide; Pola, polatuzumab vedotin

IDI and total ARDI of DXR, CPA, and Pola were compared between patients aged < 80 and ≥ 80 years. At treatment initiation, patients aged ≥ 80 years received significantly lower doses of DXR (median 59.5% vs. 100%, *p* < 0.001) and CPA (median 61.5% vs. 99.5%, *p* < 0.001) than those aged < 80 years. Pola was generally administered at or near full dose in both groups (median 95% vs. 100%, *p* = 0.012). The combined IDI of the three agents was also markedly lower in the ≥ 80 years group (67% vs. 97%, *p* < 0.001) (Table 2). Density plots illustrating IDI distributions demonstrated distinct age-related differences (Fig. 1). In patients aged < 80 years, DXR and CPA showed bimodal curves with peaks around 75% and 100%, suggesting that, though most patients received full doses, some began with modest reductions. Pola showed a unimodal Distribution peaking near 100%, indicating near-universal use of full-dose Pola at treatment initiation. In contrast, patients aged ≥ 80 years showed more pronounced dose reductions. For DXR and CPA, the Distributions shifted leftward, with bimodal peaks around 50% and 75%, indicating that initial dose reductions were common. Pola in this group retained a major peak near 100%, but also exhibited a smaller secondary peak around 50%, suggesting selective dose attenuation in a subset of patients.

**Table 2:** Initial and average relative dose intensity of chemotherapeutic agents stratified by age group

	ALL patients(N = 172)	Age <80 y(n = 128)	Age ≥80 y(n = 44)	P-value
DXR				
IDI, median (range)	93 (0-118)	100 (50-118)	59.5 (0-93)	<0.001
ARDI, median (range)	83 (0-105)	89 (12-105)	55 (0-91)	<0.001
CPA				
IDI, median (range)	90 (46-118)	99.5 (48-118)	61.5 (46-100)	<0.001
ARDI, median (range)	81 (12-103)	87 (12-103)	58 (13-89)	<0.001
Pola				
IDI, median (range)	100 (50-138)	100 (50-138)	95 (50-102)	0.012
ARDI, median (range)	88 (16-116)	89 (17-116)	84 (16-103)	0.027
Combined (DXR, CPA, Pola)				
IDI, median (range)	92 (50-125)	97 (54-125)	67 (50-91)	<0.001
ARDI, median (range)	84 (13-122)	88 (13-122)	65 (13-90)	<0.001

Abbreviations: DXR, doxorubicin; CPA, cyclophosphamide; Pola, polatuzumab vedotin; IDI, initial dose intensity; ARDI, average relative dose intensity

**Fig.2:**
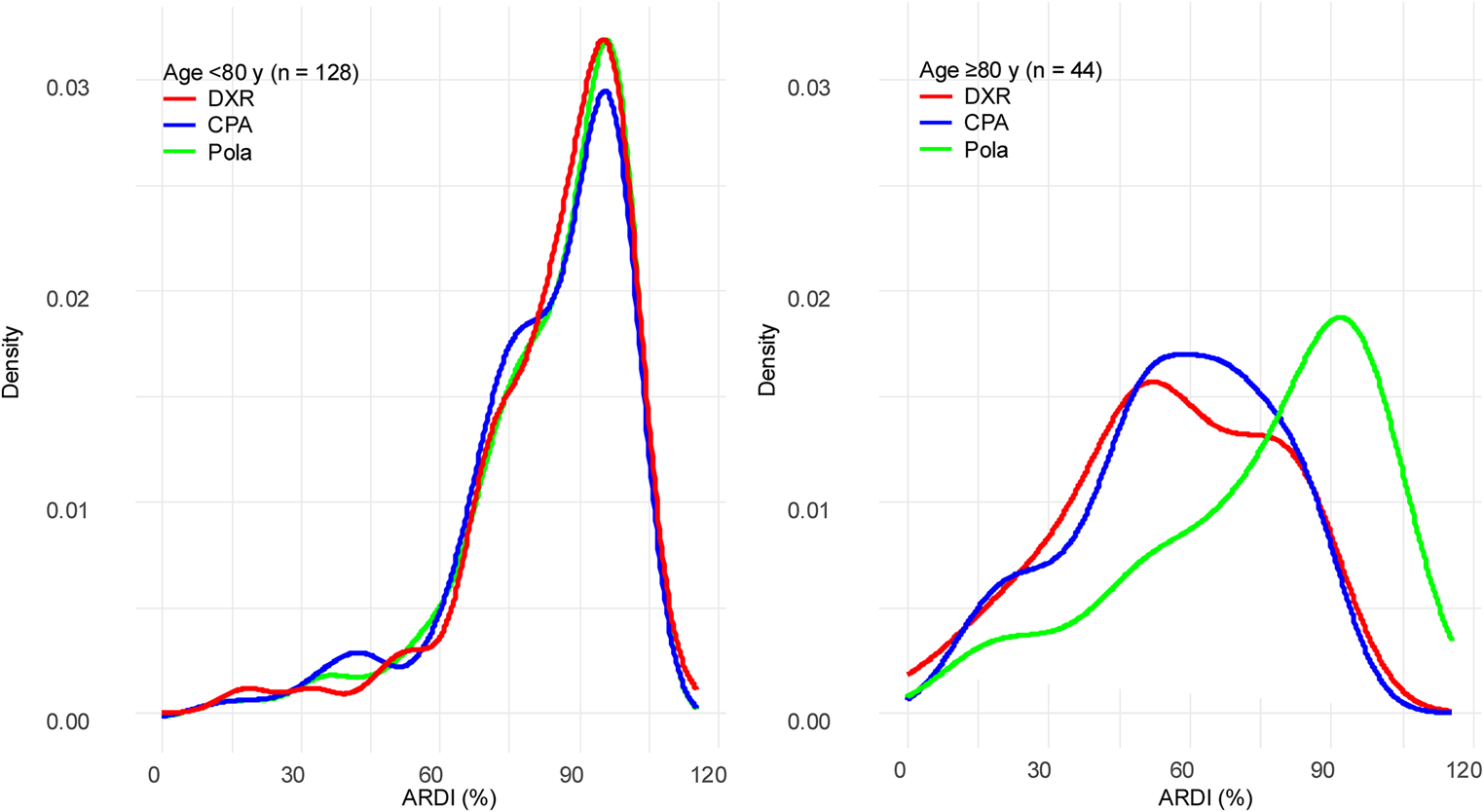
Density plots of average relative dose intensity of DXR, CPA, and Pola stratified by age group (< 80 years vs. ≥ 80 years) Kernel density plots showing the distribution of average relative dose intensity (**ARDI**, %) for doxorubicin (**DXR**), cyclophosphamide (**CPA**), and polatuzumab vedotin (**Pola**) in patients with diffuse large B-cell lymphoma. The left panel shows patients aged < 80 years (n = 128), and the right panel shows those aged ≥ 80 years (n = 44). Abbreviations: ARDI, average relative dose intensity; DXR, doxorubicin; CPA, cyclophosphamide; Pola, polatuzumab vedotin

A similar trend was observed in ARDI, which reflects dose maintenance over time (Table 2). ARDI was significantly lower in the ≥ 80 years group for both DXR (median 55% vs. 89%, *p* < 0.001) and CPA (median 58% vs. 87%, *p* < 0.001), whereas Pola maintained a relatively high ARDI (median 84% vs. 89%, *p* = 0.027). The combined ARDI for the three agents was also notably lower in the older group (65% vs. 88%, *p* < 0.001). ARDI distribution curves further highlighted these trends (Fig. 2). In patients aged < 80 years, both DXR and CPA had sharp unimodal peaks around 95%, indicating consistent dose maintenance. In the ≥ 80 years group, DXR exhibited a bimodal pattern with peaks near 50% and 75%, whereas CPA showed a broader peak centered around 55%, reflecting more heterogeneous and frequent dose attenuation. For Pola, the < 80 years group had a tight peak near 100%, with a minor shoulder at 75%, indicating occasional modest reductions. In the ≥ 80 years group, the main peak remained at 95%, but a secondary shoulder appeared near 50%, suggesting that, though high-dose maintenance was common, some patients underwent more substantial reductions over time.

To complement the kernel density plots, cumulative distribution plots of IDI and ARDI were generated (Supplementary Figures S1 and S2). The cumulative distribution of IDI demonstrated a bimodal pattern for DXR and CPA, reflecting physician heterogeneity in selecting initial doses, whereas Pola was administered at full dose in nearly all patients. In contrast, the ARDI plots showed broader distributions for DXR and CPA, indicating cumulative dose reductions during treatment, whereas Pola remained right-shifted with most patients maintaining ARDI ≥ 90%, underscoring the clinical emphasis on sustaining full dosing of the antibody-drug conjugate component.

### Treatment Efficacy

Of the 172 patients included in the study, 151 (87.8%) were evaluable for response by PET-CT at the end of treatment. The median follow-up duration at the time of data cutoff was 10.7 months (range, 0.3–25.2) months. The ORR in the evaluable cohort was 95.4% (95% CI: 90.7–97.7%), including CR in 75.5% and PR in 19.9% (Table 3). In patients aged < 80 years, the ORR was 97.3% (95% CI: 92.5–99.1%), compared with 89.5% (95% CI: 75.9–95.8%) in those aged ≥ 80 years (*p* = 0.235). The CR rate was also numerically higher in the younger group (78.8% vs. 65.8%), although the difference was not significant (*p* = 0.152).

**Table 3:** Treatment response rates stratified by age group (<80 years vs ≥80 years)

	All patients(N = 151)	Age <80 y(n = 113)	Age ≥80 y(n = 38)	P-value
ORR, n (%)[95% CI]	144 (95.4%)[90.7-97.7]	110 (97.3%)[92.5-99.1]	34 (89.5%)[75.9-95.8]	0.067
CR, n (%)[95% CI]	114 (75.5%)[68.1-81.7]	89 (78.8%)[70.3-85.3]	25 (65.8%)[49.9-78.8]	0.128
PR, n (%)[95% CI]	30 (19.9%)[14.3-26.9]	21 (18.6%)[12.5-26.7]	9 (23.7%)[13.0-39.2]	0.489

Abbreviations: ORR, overall response rate; CR, complete response; PR, partial response; CI, confidence interval.Footnote: At the time of data cutoff, after a median follow-up of 10.7 (range 0.3-25.2) months, 151 patients were evaluated for efficacy by PET-CT at the end of treatment

To explore factors associated with treatment response, multivariable logistic regression analysis was performed (Table 4). In the overall cohort, no variables, including patient sex, average IDI of cytotoxic agents (DXR and CPA), IDI of Pola, GNRI, and IPI score, showed significant associations with response. However, in the subgroup of patients aged ≥ 80 years, the GNRI was independently associated with higher odds of response (OR 1.09, 95% CI: 1.00–1.19, *p* = 0.045), suggesting that nutritional status may play a critical role in treatment efficacy in very elderly patients.

**Table 4:** Multivariable logistic regression analysis of factors associated with treatment response

All patients (N = 151)	OR	95% CI	P-value
Male	0.589	0.25-1.37	0.22
Average IDI of DXR and CPA	1.01	0.98-1.05	0.38
Average IDI of Pola	1.01	0.98-1.05	0.41
GNRI	1.04	0.99-1.08	0.07
IPI score	0.777	0.54-1.11	0.17
Age <80 y (n = 113)	OR	95％ CI	P-value
Male	0.761	0.28-2.04	0.59
Average IDI of DXR and CPA	1.01	0.95-1.06	0.84
Average IDI of Pola	1.01	0.96-1.07	0.7
GNRI	1.01	0.97-1.06	0.55
IPI score	0.674	0.44-1.03	0.069
Age ≥80 y (n = 38)	OR	95％ CI	P-value
Male	0.337	0.05-2.23	0.26
Average IDI of DXR and CPA	1.03	0.96-1.11	0.43
Average IDI of Pola	1.02	0.97-1.08	0.46
GNRI	1.09	1.00-1.19	0.045
IPI score	0.835	0.34-2.06	0.7

Abbreviations: OR, odds ratio; CI, confidence interval; IDI, initial dose intensity; DXR, doxorubicin; CPA, cyclophosphamide; Pola, polatuzumab vedotin; GNRI, Geriatric Nutritional Risk Index; IPI, International Prognostic Index

In an additional subgroup analysis focusing on elderly patients, outcomes were compared between those aged 65–79 years and those aged ≥ 80 years (Supplementary Table S2). The ORR was similar between the two subgroups, whereas the CR rate was numerically lower in the ≥ 80 years group.

### Safety and Adverse Events

The incidence of grade ≥ 3 AEs and TRM is summarized in Table 5. Overall, 45.3% of patients experienced non-hematological severe AEs. The incidence was comparable between patients aged < 80 years and those aged ≥ 80 years (42.9% vs. 52.3%, *p* = 0.298). The most common severe AEs were febrile neutropenia (*n* = 20), pneumonia (*n* = 15), and COVID-19 infection (*n* = 15), followed by decreased appetite (*n* = 12), sepsis/bacteremia (*n* = 5), and elevated transaminases (*n* = 5)(Supplementary Table S1). Regarding hematopoietic growth factor support, daily filgrastim was administered to 166 of 172 patients (96.5%), and pegfilgrastim was administered to 84 of 172 patients (48.8%). The incidence of severe neutropenia was numerically higher in the ≥ 80 years group (43.2% vs. 63.3%), but the difference was not significant (*p* = 0.162). Similarly, no significant differences were observed between age groups in the incidence of grade ≥ 3 anemia or thrombocytopenia. However, TRM was significantly higher in patients aged ≥ 80 years than in those aged < 80 years (11.4% vs. 2.3%, *p* = 0.027). The causes of TRM in the older group included pneumonia (*n* = 2), COVID-19 (*n* = 1), sepsis (*n* = 1), and stroke (*n* = 1).

**Table 5:** Incidence of grade ≥3 adverse events and treatment-related mortality stratified by age group

	ALL patients(N = 172)	Age <80 y(n = 128)	Age ≥80 y(n = 44)	P-value
Anemia, n (%)	29 (16.9)	22 (17.2)	7 (15.9)	0.557
Neutropenia, n (%)	100 (58.1)	81 (63.3)	19 (43.2)	0.162
Thrombopenia, n (%)	13 (7.6)	9 (7.0)	2 (4.5)	0.906
Severe AE, n (%)	78 (45.3)	55 (42.9)	23 (52.3)	0.298
TRM, n (%)	8 (4.7)	3 (2.3)	5 (11.4)	0.027

Abbreviations: AE, adverse event; TRM, treatment-related mortality.Footnote: All adverse events listed were grade ≥3 according to the Common Terminology Criteria for Adverse Events. The causes of death in patients aged ≥80 years were pneumonia (n = 2), COVID-19 (n = 1), sepsis (n = 1), and stroke (n = 1)

To identify predictors of severe AEs, a multivariable logistic regression analysis was performed (Table 6). Of the candidate variables, higher IDI of Pola (OR 1.05, 95% CI: 1.01–1.09, *p* = 0.025) and lower GNRI (OR 0.949, 95% CI: 0.914–0.986, *p* = 0.007) were significantly associated with an increased risk of severe AEs. Age ≥ 80 years, sex, performance status, and average IDI of DXR and CPA were not significant predictors.

**Table 6:** Multivariable logistic regression analysis of factors associated with severe adverse events

	OR	95% CI	P-value
Age (≥80 y)	1.55	0.491-4.900	0.455
Male	1.34	0.630-2.850	0.447
PS (score)	1.14	0.755-1.730	0.531
Average IDI of DXR and CPA	0.992	0.960-1.020	0.625
IDI of Pola	1.05	1.010-1.090	0.025
GNRI	0.949	0.914-0.986	0.007

Abbreviations: OR, odds ratio; CI, confidence interval; PS, performance status; IDI, initial dose intensity; DXR, doxorubicin; CPA, cyclophosphamide; Pola, polatuzumab vedotin; GNRI, Geriatric Nutritional Risk Index

When elderly patients were further stratified into 65–79 years and ≥ 80 years groups, the incidence of SAEs was comparable between the two subgroups (Supplementary Table S3). However, TRM was higher in the ≥ 80 years group (11.4% vs. 2.2%), with infection-related deaths being more frequent in this subgroup.

## Discussion

 This multicenter, retrospective study evaluated the real-world use of Pola-R-CHP in patients aged ≥ 80 years with newly Diagnosed DLBCL. The findings demonstrated that Pola-R-CHP is a promising treatment option in this very elderly population, achieving an ORR of 89.5%, which is comparable to the 97.3% observed in patients aged < 80 years. However, TRM was significantly higher in the ≥ 80 years group (11.4% vs. 2.3%), underscoring the importance of careful toxicity management in this age group. Further stratification within the elderly cohort demonstrated that patients aged ≥ 80 years had higher TRM than those aged 65–79 years, primarily due to infection-related deaths. These findings indicate that patients aged ≥ 80 years represent a particularly vulnerable subgroup, even among elderly individuals, and may require intensified infection prophylaxis, closer monitoring, and more cautious dose adjustments. In the present analysis, the bimodal distributions of IDI and ARDI indicate heterogeneity in prescribing behavior, which may reflect varying levels of physician comfort with initial dose attenuation and subsequent dose adjustments. In particular, in patients aged ≥ 80 years, the IDI Distributions for doxorubicin and cyclophosphamide showed Distinct peaks around 50% and 80%, suggesting two prevailing strategies: one favoring substantial upfront dose reduction in very elderly or frail patients, and the other initiating treatment closer to standard dosing when tolerance was anticipated. This heterogeneity highlights differences in physician decision-making and underscores the need for more standardized approaches to initial dosing in this vulnerable population. Although the difference in IDI of Pola between subgroups was significant (95% vs. 100%), the absolute difference was small and is unlikely to be clinically meaningful. These findings suggest that, in real-world practice, physicians generally aim to maintain full dosing of Pola even in very elderly patients, reflecting the perceived importance of sustaining exposure to the antibody drug-conjugate component.

Given the high incidence of severe neutropenia and TRM from infections observed in the study cohort, the present findings underscore the critical importance of proactive infection management. As recommended for patients receiving anthracycline-based regimens, primary prophylaxis with long-acting granulocyte colony-stimulating factor should be strongly considered to mitigate febrile neutropenia. In addition, prophylaxis against opportunistic infections, such as trimethoprim-sulfamethoxazole for *Pneumocystis jirovecii* pneumonia and acyclovir or valacyclovir for herpes simplex virus and varicella-zoster virus infections, should be standard supportive care in this vulnerable population [13]. Although data on antimicrobial prophylaxis and neurotoxicity management were not collected in this study, nearly all patients received G-CSF support, with daily filgrastim in 96.5% and pegfilgrastim in 48.8% of cases. These findings highlight the reliance on G-CSF as a cornerstone of supportive care in this population, while underscoring the need for future studies to incorporate more comprehensive supportive care data to fully assess their impact on treatment outcomes.

Notably, though the doses of DXR and CPA were substantially lower in the older cohort, the dose intensity of Pola was relatively maintained. This dosing pattern suggests that clinicians may prioritize preserving Pola dosing due to its good efficacy profile, even in very elderly patients. The present findings are consistent with previous real-world studies from Japan that examined reduced-dose Pola-R-CHP regimens in very elderly patients. Sekiguchi et al. reported outcomes for 11 patients aged ≥ 80 years who were treated with a reduced-dose Pola-R-mini-CHP regimen, achieving an ORR of 100% with no TRM [6]. Similarly, Sato et al. analyzed 38 patients aged > 80 years receiving reduced-dose Pola-R-CHP and reported 12-month overall and progression-free survival rates of 86.2% and 78.5%, respectively [7]. These findings collectively support the feasibility and efficacy of Pola-R-CHP in this age group when appropriately dose-adjusted.

Anthracycline-induced cardiotoxicity is a well-recognized concern in elderly patients with DLBCL. Although the median cumulative dose of doxorubicin in the entire cohort approached 300 mg/m², this value does not reflect uniform exposure across age groups. In fact, the ARDI of doxorubicin was lower in the elderly group: the median ARDI was 89% in patients aged < 80 years and 55% in those aged ≥ 80 years. These findings suggest that clinicians often implemented dose attenuation strategies in very elderly patients to mitigate toxicity risk. Nonetheless, even with dose reductions, elderly patients may remain at increased risk for cardiotoxicity. Therefore, a proactive cardio-oncology approach involving close collaboration with cardiologists is important. This should include baseline and serial cardiac function monitoring with echocardiography, assessing not only left ventricular ejection fraction (LVEF), but also the more sensitive global longitudinal strain (GLS). Early intervention with cardioprotective drugs, such as angiotensin-converting enzyme inhibitors or beta-blockers, should be considered in collaboration with cardiologists to mitigate cardiac damage [14, 15].

In comparison, the R-mini-CHOP regimen, developed specifically for patients aged ≥ 80 years, demonstrated an ORR of 78% and a TRM rate of 8% in a multicenter, phase II trial [16]. Whereas R-mini-CHOP remains a widely accepted treatment option for elderly DLBCL patients, the addition of Pola may provide enhanced efficacy without excessive toxicity if carefully managed. Recently, a randomized, phase III trial comparing Pola-R-miniCHP and R-miniCHOP in patients aged ≥ 80 years was presented at the European Hematology Association Congress. Interim results showed no significant difference in the incidence of severe AEs between the two regimens [17], suggesting that Pola-R-miniCHP may be a safe and feasible alternative to R-miniCHOP in selected patients, although mature efficacy data are still awaited.

An important consideration is the optimal treatment duration for patients with limited-stage Disease. Though a shorter course of 3–4 cycles of immunochemotherapy followed by radiotherapy is often considered standard for this group, this approach is primarily supported by evidence from studies of the R-CHOP regimen [18, 19]. Notably, the pivotal POLARIX trial administered six cycles of Pola-R-CHP plus two cycles of rituximab to all patients, including those with limited-stage disease [5]. Whether the evidence supporting abbreviated cycles in the R-CHOP era is applicable to the more potent Pola-R-CHP regimen remains unclear. In the present real-world cohort, the decision to administer six cycles likely reflects adherence to the POLARIX protocol, pending further data from ongoing or future studies exploring abbreviated regimens with Pola-R-CHP.

Furthermore, though patient selection based on biological characteristics is important, the socioeconomic aspect of treatment is also a critical consideration. The use of Pola-R-CHP warrants careful evaluation of its cost-effectiveness, particularly in light of findings from the POLARIX trial. Although this trial demonstrated a significant improvement in PFS in the overall population, a pre-specified subgroup analysis suggested that the benefit may be more pronounced in patients with the activated B-cell like subtype, as classified by gene expression profiling [5]. Given that Pola-R-CHP is substantially more expensive than R-CHOP, several health economic evaluations have assessed its value. A recent cost-effectiveness analysis from the perspective of the Japanese public healthcare system concluded that Pola-R-CHP is cost-effective [20]. A similar study in the United States reached comparable conclusions [21]. These findings suggest that, although the upfront drug cost is high, the long-term benefits, such as improved survival and potential reduction in relapse-related costs, justify its use from a health economics standpoint. Therefore, in addition to biological subtyping, our real-world experience supports the consideration of Pola-R-CHP in very elderly patients from both clinical and economic perspectives.

Importantly, the present study provides a detailed analysis of the impact of initial and cumulative dose intensities, specifically IDI and ARDI, on clinical outcomes in this population. Whereas Sato et al. previously reported ARDI patterns in this population [7], the present findings further clarify how dose intensity affects efficacy and safety in very elderly patients. The present results reinforce the clinical importance of maintaining adequate ARDI in elderly patients, which has also been highlighted in previous reports, even in those aged ≥ 80 years [22]. Notably, a higher IDI of Pola was associated with an increased incidence of severe AEs. This may be explained by the pharmacological mechanism of Pola, an antibody-drug conjugate (ADC) targeting CD79b that delivers monomethyl auristatin E (MMAE) into malignant B cells [23]. Following internalization, MMAE disrupts microtubules and induces apoptosis, but this effect may extend to off-target tissues and contribute to toxicity, particularly in physiologically vulnerable individuals. Similar toxicity profiles have been reported with brentuximab vedotin, another ADC carrying MMAE, which has been associated with peripheral neuropathy and hematological toxicities in older populations [24]. These findings highlight the need for thoughtful dose selection and close toxicity monitoring when using Pola in very elderly patients.

Furthermore, the present study identified the GNRI as an independent predictor of both treatment response and the occurrence of severe AEs in patients treated with Pola-R-CHP. This finding underscores the clinical significance of assessing nutritional status in very elderly DLBCL patients. Notably, previous research has demonstrated that the combination of GNRI with the Charlson Comorbidity Index enhances prognostic accuracy for overall survival in elderly DLBCL patients, surpassing the predictive power of either index alone [25]. These insights suggest that integrating nutritional and comorbidity assessments can provide a more comprehensive evaluation of patient risk profiles. In addition to nutritional and comorbidity evaluations, geriatric assessment (GA) has emerged as a valuable tool in the management of elderly oncology patients [26]. GA encompasses evaluations of functional status, comorbidities, cognition, psychological state, social support, and nutritional status, offering a multidimensional approach to patient assessment. Studies have shown that GA can predict chemotherapy-related toxicities and overall survival in elderly DLBCL patients [27]. Implementing GA in clinical practice may facilitate individualized treatment strategies and optimize therapeutic outcomes while minimizing adverse effects in this vulnerable population.

Despite these insights, the present study has several limitations. The retrospective design may introduce selection bias, and treatment decisions, including dose modifications, were at the discretion of the treating physicians. Information on patients who discontinued treatment prematurely or did not undergo end-of-treatment PET evaluations was also lacking, precluding assessment of the impact of early discontinuation on outcomes. In addition, the follow-up period was relatively short, limiting our ability to assess long-term outcomes such as progression-free and overall survivals. Future prospective studies with longer follow-up are needed to validate the present findings and to develop optimized treatment strategies that incorporate frailty assessments and nutritional evaluations for very elderly DLBCL patients.

## Conclusion

 Pola-R-CHP demonstrates high efficacy for DLBCL in patients aged ≥ 80 years, but it is associated with significant TRM. The GNRI was an independent predictor of both treatment response and severe adverse events. Based on these findings, individualized approaches, incorporating careful dose modification and nutritional assessment, are considered essential for optimizing the balance between efficacy and safety in this vulnerable population.

## Supplementary Information

Below is the link to the electronic supplementary material.

Supplementary Material 1 (XLSX. 9.76 KB)

Supplementary Material 2 (XLSX 10.8 KB)

Supplementary Material 3 (XLSX 10.4 KB)

Supplementary Material 4 (DOCX. 181 KB)

## Data Availability

The data that support the findings of this study are not publicly available due to their containing information that could compromise the privacy of research participants but are available from the corresponding author upon reasonable request.
