# Supplementary material for: Polatuzumab vedotin, rituximab, cyclophosphamide, doxorubicin, and prednisone (Pola-R-CHP) therapy in diffuse large B-cell lymphoma in patients aged 80 years or older: a real-world study
Source: Ann Hematol. 2025 Oct 10;104(10):5191–200. doi: 10.1007/s00277-025-06619-0 (PMC12619725; doi:10.1007/s00277-025-06619-0)
Supplement: Supplementary file 4 — Supplementary Material 4 (DOCX. 181 KB) [file 277_2025_6619_MOESM4_ESM.docx]

**Supplementary Fig. S1 Cumulative distribution plots of initial dose intensity for DXR, CPA, and Pola**

Doxorubicin (DXR) and cyclophosphamide (CPA) show bimodal distributions, reflecting heterogeneity in initial dosing decisions, whereas polatuzumab vedotin (Pola) is administered at full dose in nearly all patients.

Abbreviations: DXR, doxorubicin; CPA, cyclophosphamide; Pola, polatuzumab vedotin.

**Supplementary Fig. S2 Cumulative distribution plots of average relative dose intensity for DXR, CPA, and Pola.**

Doxorubicin (DXR) and cyclophosphamide (CPA) demonstrate broader distributions, indicating cumulative dose reductions during therapy. In contrast, polatuzumab vedotin (Pola) remains right-shifted, with most patients maintaining ARDI ≥90%, highlighting the clinical priority of maintaining full exposure.

Abbreviations: ARDI, average relative dose intensity; DXR, doxorubicin; CPA, cyclophosphamide; Pola, polatuzumab vedotin.
